# WRINKLED1 Is Subject to Evolutionary Conserved Negative Autoregulation

**DOI:** 10.3389/fpls.2019.00387

**Published:** 2019-03-28

**Authors:** Per Snell, Åsa Grimberg, Anders S. Carlsson, Per Hofvander

**Affiliations:** Department of Plant Breeding, Swedish University of Agricultural Sciences, Alnarp, Sweden

**Keywords:** self-regulated gene, gene circuit, gene regulation, intrinsically disordered region, genetic engineering, protein engineering, fluorescent electrophoretic mobility shift assay

## Abstract

High accumulation of storage compounds such as oil and starch are economically important traits of most agricultural crops. The genetic network determining storage compounds composition in crops has been the target of many biotechnological endeavors. Especially WRINKLED1 (WRI1), a well-known key transcription factor involved in the allocation of carbon into oil, has attracted much interest. Here we investigate the presence of an autoregulatory system involving WRI1 through transient expression in *Nicotiana benthamiana* leaves. Different lengths of the Arabidopsis *WRI1* promotor region were coupled to a GUS reporter gene and the activity was measured when combined with constitutive expression of different WRI1 homologs from *Arabidopsis thaliana*, oat (*Avena sativa* L.), yellow nutsedge *(Cyperus esculentus* L.), and potato (*Solanum tuberosum* L.). We could show that increasing levels of each WRI1 homolog reduced the transcriptional activity of the Arabidopsis *WRI1* upstream region. Through structural analysis and domain swapping between oat and Arabidopsis WRI1, we were able to determine that the negative autoregulation was clearly dependent on the DNA-binding AP2-domains. A DNA/protein interaction assay showed that AtWRI1 is unable to bind to its corresponding upstream region indicating non-direct interaction *in vivo*. Taken together, our results demonstrate a negative feedback loop of *WRI1* expression and that it is an indirect interaction most likely caused by downstream targets of WRI1. We also show that it is possible to release *WRI1* expression from this autoregulation by creating semi-synthetic WRI1 homologs increasing the potential use of WRI1 in biotechnological applications.

## Introduction

During seed maturation, many plants accumulate triacylglycerol (TAG) together with starch and storage proteins in their seeds to be used as energy reserves for the germinating seedling (Athenstaedt and Daum, 2006). The sequestration of carbon to energy-dense storage molecules, such as starch and TAG, in seeds, requires both a shift of the carbon flow from source tissues into newly established sink tissue and an allocation of carbon within the sink into the synthesis of specific storage molecules. In Arabidopsis, carbon is initially stored as starch in developing seeds which is later degraded and remobilized into TAG biosynthesis (Ruuska et al., 2002). This process is analogous to the seed development in the oil crop rapeseed (*Brassica napus* L.) where starch also is utilized for short-term carbon storage before allocation of carbon into TAG (Kang and Rawsthorne, 1994). Both TAG and starch are synthesized by redirecting carbon away from the main glycolysis pathway but very little is known of why plants utilize starch as a carbon stockpile instead of incorporating it directly into TAG. Although we do have extensive knowledge of the separate/individual biosynthetic pathways of both starch and TAG, we have little understanding of the genetic regulatory system behind the switch between starch and TAG accumulation.

Spatial and temporal variation in gene expression in eukaryotes involves a multitude of regulatory mechanisms where one of the earliest and arguably the most important is transcriptional regulation. Transcriptional regulation in plants, especially the early steps of initiation and promoter escape, is commonly tightly controlled through the binding of general and sequence-specific transcription factors (TFs) to the regulatory elements located within the genomic regulatory regions. Sequence-specific TFs usually target cis-regions (non-protein coding DNA regions) often located upstream controlling sets of genes. TFs generally function as either activators or repressors when interacting with a specific regulatory element and where TFs can act as both a repressor and an activator on the same regulatory element (Torti and Fornara, 2012; Fernandez et al., 2014).

WRINKLED1 (WRI1, At3g54320) is an APETALA2/ethylene-responsive element binding protein (AP2/EREBP) TF involved in regulating the expression of genes important for carbon allocation into TAG in plants (Cernac and Benning, 2004). It was first described in Arabidopsis where the loss-of-function mutation of *WRI1* results in up to 80% reduction in Arabidopsis seed oil content and a 50% increase in sucrose (Focks and Benning, 1998). WRI1 has been shown to orchestrate the regulation of a number of genes involved in shuffling carbon from sucrose and starch into fatty acid synthesis through glycolysis (Baud et al., 2007; Maeo et al., 2009). Recently, it was revealed that this pattern of regulation has been preserved not only through the evolution of plants but also between different plant tissues indicating that WRI1 does not require a seed environment to function (Grimberg et al., 2015). Functional WRI1 orthologs have been identified in a number of different higher plant species such as rapeseed, maize (*Zea mays* L.), potato (*Solanum tuberosum* L.), yellow nutsedge (*Cyperus esculentus* L.), and oat (*Avena sativa* L.) (Liu et al., 2010; Shen et al., 2010; Grimberg et al., 2015). WRI1 contains two AP2 DNA binding domains enabling WRI1 to bind to the AW-box ([CnTnG](n)_7_[CG]) located in the promoter region of downstream target genes thereby regulating their expression (Cernac and Benning, 2004; Maeo et al., 2009). Furthermore, WRI1 has a total of three intrinsically disordered regions (IDRs), one located upstream of the two AP2 domains while the other two are found downstream. IDR3 has been shown to be important for the *in vivo* stability of Arabidopsis WRI1 through the presence of a PEST-motif located within it (Ma et al., 2015). Recently, it was discovered that SUCROSE-NON-FERMENTING-1-RELATED PROTEIN 19 KINASE-1 (SnRK1) influences the turnover rate of WRI1 by phosphorylating within the tandem AP2-domains leading to increased proteasomal degradation (Zhai et al., 2017). It has also been shown that trehalose 6-phosphate interacts with subunits of the SnRK1-complex leading to reduced phosphorylation of Arabidopsis WRI1 and thereby longer half-life (Zhai et al., 2018).

Many TFs are known to not only regulate expression of other genes but also its own expression, a mechanism commonly referred to as autoregulation. This is especially common in prokaryotes such as the *Escherichia coli* strain K12 in which more than half of all TFs are autoregulated and negative autoregulation appears to be most common (Stekel and Jenkins, 2008). The number of TFs under autoregulation shows that it is an evolutionary favored system although we have yet to understand the main purpose of such regulatory loops. An extensive amount of work has been performed using mathematical modeling to try to understand these systems and there are two main explanatory models. The first suggests that negative autoregulation can be used to maintain specific levels of TFs (Becskei and Serrano, 2000; Wall et al., 2003; Shinar et al., 2006; Fournier et al., 2007). The other proposes that negative autoregulation reduces the response time for a gene to a signal (Rosenfeld et al., 2002). Autoregulatory circuits have been discussed and utilized in synthetic biology and the increased utilization of multi-gene constructs and modification of regulatory elements in plant biotechnology calls for an increased knowledge of the mechanisms behind autoregulation.

This study reveals that one of the most commonly used genes for biotechnological modification of plant oil biosynthesis, *WRI1*, is under an evolutionary conserved negative autoregulation. In addition to this, it is also shown that domain swapping between different WRI1 homologs could reduce the impact of this negative autoregulation making it a potential approach for the production of high-oil yielding crops.

## Materials and Methods

### Plant Material

All plant material was grown in controlled growth chambers (Biotron, SLU Alnarp, Sweden) under either fluorescent lights or metal-halide lamps with 200 μmol ⋅ m^-2^ ⋅ s^-1^ and with a 15.5/8.5 h (light/dark) photoperiod. The temperature was set to 25/20°C (light/dark) and a relative humidity of 60%.

### Binary Vector Construction

To allow for easy assembly of the upstream region to the GUS gene the pSCV1.6, a binary vector containing a CaMV 35S driven nptII gene together with a CaMV 35S driven gusA, was modified by replacing the CaMV 35S-promoter with a Gateway^®^cassette. The conversion was carried out in accordance to the manufacturer’s instructions using Gateway^®^Vector Conversion System (Life Technologies, Carlsbad, CA, United States) and with SmaI being used to excise the CaMV 35S promoter and hereafter referred to as GW::GUS. The GW::GUS construct was sequenced to ensure correct insertion of the Gateway^®^cassette.

### Cloning of Genes and Upstream Regions

Isolation and cloning of Arabidopsis *WRI1* and its homologs have previously been described (Grimberg et al., 2015) but in short were oat and yellow nutsedge *WRI1* isolated from cDNA-libraries prepared from grain endosperm and tuber parenchyma, respectively. Arabidopsis and potato *WRI1* were synthesized (Eurofins, Ebersberg, Germany) based on sequence data from openly available resources. Isolated genes were then cloned into binary vectors (pK2GW7, pART27, or pXZP393) and transformed into *Agrobacterium tumefaciens* (strain GV3101mp90) using electroporation for later use in agroinfiltration. Binary plasmids containing *p19* and *GFP* were kindly provided by Wood et al. (2009). Permutations of *WRI1* were produced by a PCR-based fusion protocol described by Shevchuk et al. (2004). Identification of WRI1 homologs in oat, yellow nutsedge, and potato was done by sequence similarity to Arabidopsis WRI1 and verified using functional analysis in *Nicotiana benthamiana* (Grimberg et al., 2015). All WRI1 encoding sequences were under expressional control of a CaMV 35S promoter.

The 2000 bp upstream region for Arabidopsis *WRI1* was isolated from gDNA using primers designed using sequence data from TAIR9 for region Chr3:2012807-20114807 and cloned into GW::GUS using the Gateway^®^cloning system in accordance with the manufacturer’s instructions. All three additional variations in length of the Arabidopsis *WRI1* upstream region were created in a similar manner with the exception that the isolated 2000 bp region was used as starting material instead of gDNA.

### Transient Gene Expression in *N. benthamiana* Leaves

Agroinfiltration of *N. benthamiana* leaves for transient gene expression were conducted in accordance with Wood et al. (2009) and Grimberg et al. (2015) with the exception that two leaves of similar developmental age were used on each plant and that sampling was done after 4 days. For GUS-activity assays, two culture mixtures were infiltrated in each leaf with the main leaf vein being used for separation of the infiltrated areas. Leaf tissue for visualization of GUS-activity was sampled using a hole puncher (Ø 8 mm). For lipid analysis and GUS-quantification, approximately 800 and 100 mg, respectively, of leaf tissue were sampled and flash frozen in liquid nitrogen and stored at -80°C until further use.

### Lipid Extraction and Isolation

Lipid analyses were carried out on freeze-dried leaf material by first extracting total lipids using a modified Bligh and Dyer method (Bligh and Dyer, 1959). Samples were homogenized in glass Potter-Elvehjem tissue homogenizers in 3.75 ml MeOH:ChCl_3_ (2:1, v.v) and 1 ml of 0.15 M acetic acid and transferred to a glass tube with a Teflon lined screw cap. The homogenizer was rinsed using 1.25 ml of chloroform which was added to the sample before 1.25 ml of water was added and the sample thoroughly mixed by vortexing. The samples were centrifuged at 400 × *g* for 2 min and the chloroform phase was transferred to a new screw cap tube using glass Pasteur pipettes. Total lipid extracts corresponding to 10 mg dry weight were evaporated until dry at ∼98°C under continuous N_2_ flow. The samples were resuspended in 10 μl chloroform before being loaded onto a silica gel TLC-plate (Silica 60; Merck). The lipids were separated in heptane:diethyl ether:acetic acid (70:30:1, v.v.v) and the plate stained with primulin to visualize lipids under UV-light. Lipid species were identified using migratory pattern in comparison with authentic lipid standards and the silica gel was scraped off after being sprayed with water. The silica gel was transferred to new screw cap tubes and 2 ml dry MeOH was added and evaporated at ∼96°C under continuous N_2_ flow. Methylation of lipids was done by adding 2 ml of 2% H_2_SO_4_ in methanol and incubating the samples at 90°C for 45 min. The samples were allowed to cool to room temperature before 168 nmol of heptadecanoic acid methyl ester was added as internal standard and the fatty acid methyl esters (FAMEs) was extracted by adding 1 ml hexane and 2 ml water followed by centrifugation at 400 × *g* for 2 min. The hexane phase was transferred to an evaporation tube using a glass Pasteur pipette and the hexane was evaporated by gentle heating under continuous N_2_ flow. The samples were resuspended in 300 μl hexane and the FAME composition analyzed by gas-liquid chromatography (GLC).

### Gas-Liquid Chromatography

All GLC analyses were conducted on a Shimadzu-GLC 17A (Shimadzu, Kyoto, Japan) equipped with a 50 m CP-Wax 58 column (ID: 0.32 mm) with hydrogen as a carrier gas (linear velocity: 75.6 cm/s). Detection of FAMEs was done by a flame ionization detector. For separation, a temperature program was used where the initial temperature was set to 150°C with a final temperature of 210°C (ramp rate: 4°C/min) followed by an increase to 250°C with a ramp rate of 10°C/min; 2 μl of sample was injected using split injection mode with a injector temperature of 250°C.

Fatty acid methyl esters were identified using authentic standards. Peak integration and quantification were done using Shimadzu CLASS VP Version 4.3 (Shimadzu, Kyoto, Japan) software and the internal standard as a reference.

### GUS-Activity Assay

For GUS-visualization, leaf discs were vacuum infiltrated (2 × 2 min, -600 mbar) in GUS-staining solution [0.1 M NaH2PO4, 0.5 mM K3(Fe(CN)6), 0.5 mM K4(Fe(CN)6), 10 mM Na2EDTA, 2.4 mM X-Gluc, and 0.1 % Triton X-100] before being incubated at 37°C for approximately 17 h. Leaf discs were then de-stained using 95% ethanol.

### GUS-Quantification

GUS-expression was quantified by grinding 100 mg of flash frozen leaf tissue before 200 μl of GUS-extraction buffer (50 mM NaH2PO4, 10 mM EDTA, 0.1% SDS, 0.1% Triton X-100, 10 mM β-mercaptoethanol) was added and the samples were vortexed individually until thawed. After vortexing, the samples were centrifuged at 12,000 × *g* for 20 min and the supernatant was carefully transferred to new tubes. The total protein content was determined using Pierce^TM^ BCA Protein Assay Kit – Reducing Agent Compatible (Thermo Fisher Scientific, Waltham, MA, United States) in accordance with the manufacturer’s instructions; 20 μl of protein extract (corresponding to between 10 and 50 μg of protein) was added to 980 μl of pre-warmed reaction buffer (1 mM 4-methylumbelliferyl (4-MU)-β-D-glucuronide hydratein GUS-extraction buffer) and incubated at 37°C. The reaction was stopped by addition of 800 μl stop solution (0.2 M Na2CO3) to 200 μl at time points 0, 5, 15, 30, and 60 min. Fluorescence was measured using a Fluoroskan Ascent FL (Thermo Fisher Scientific, Waltham, MA, United States, excitation wavelength 365 nm, emission wavelength 455 nm, filter wavelength 430 nm) and 4-MU was quantified using a standard curve and GUS-activity was calculated as pmol 4-MU/min/mg total protein.

### Bioinformatic Analysis

Intrinsically disordered regions were predicted using the PONDR^®^tool^1^ using the PONDR^®^VL3-BA (Radivojac et al., 2003) predictor on full length translated protein sequences (Supplementary Table S2). Prediction of PEST motifs was done using ePESTfind^2^ using standard threshold score (+5.0).

### Cloning and Expression of AtWRI1

Coding sequences of *AtWRI1* without start codon was cloned into pEZY19 (Guo et al., 2008) [pEZY19 was a gift from Yu-Zhu Zhang (Addgene plasmid # 18668)] using the Gateway^®^cloning system in accordance with the manufacturer’s instructions. The decahistidine tagged *AtWRI1* (His-AtWRI1) was transformed into chemically competent Rosetta^TM^ (Merck, Darmstadt, Germany) host strain in accordance with the manufacturer’s instructions. Due to the protein being aggregated into inclusion bodies, an on column refold approach using a low-pressure chromatography system was used. For expression, 200 ml of culture [lysogeny broth with carbenicillin (50 μg/ml)] was grown at 37°C until OD_600_ ∼ 0.6. Expression was induced by 1 mM isopropyl β-D-1-thiogalactopyranoside (IPTG) for 4 h at 37°C before cells were harvested by centrifugation (4000 × *g*, 20 min). The cells were resuspended in 50 ml of 20 mM sodium phosphate buffer (pH 7.4) and centrifuged again. The cells were resuspended in 20 ml disruption buffer [20 mM sodium phosphate buffer (pH 7.4), cOmplete^TM^ Protease Inhibitor Cocktail (Merck, Darmstadt, Germany)] and disrupted mechanically using 0.5 mm zirconia/silica and a FastPrep^®^-24 Classic (MP Biomedicals, Santa Ana, CA, United States) (10 × 30 s, 4°C). The lysate was centrifuged at 15,000 × *g* for 10 min at 4°C and the pellet resuspended in protein wash buffer (20 mM sodium phosphate buffer, 2 M urea, 0.5 M NaCl, 2% Triton X-100, pH 7.4) and centrifuged again. The pellet was resuspended in denaturing solubilization buffer (20 mM sodium phosphate buffer, 8 M urea, 0.5 M NaCl, 5 mM imidazole, 2% Triton X-100, pH 7.4) and incubated at room temperature and under stirring for 60 min before being centrifuged at 15,000 × *g* for 15 min and the supernatant filtrated through a 0.45 μm filter. The denatured His-AtWRI1 was bound to a 1 ml HisTrap^TM^ HP column (Amersham Bioscience), washed with solubilization buffer, and refolded using a linear gradient from solubilization buffer to refolding buffer (20 mM sodium phosphate buffer, 0.5 M NaCl, 20 mM imidazole, pH 7.4) over 30 ml. Bound His-AtWRI1 was eluted using elution buffer (20 mM sodium phosphate, 0.5 M NaCl, 500 mM imidazole, pH 7.4). The protein containing fractions were identified by SDS-PAGE (ClearPAGE, C.B.S. Scientific, San Diego, CA, United States) and desalted using a PD10 column (GE Healthcare, Chicago, IL, United States) in accordance with respective manufacturer’s instructions. Protein concentration was measured using Pierce^TM^ BCA Protein Assay Kit (Thermo Fisher Scientific, Waltham, MA, United States) in accordance with the manufacturer’s instructions.

### Fluorescent Electrophoretic Mobility Shift Assay (fEMSA)

DNA binding reactions for the fluorescent electrophoretic mobility shift assay (fEMSA) were carried out in 150 mM KCl, 0.1 mM dithiothreitol, 10 mM Tris, 150 ng of LightShift^TM^ Poly (dI-dC) (Thermo Fisher Scientific, Waltham, MA, United States), and 50 ng CY5-labeled probe DNA at pH 7.4 in a total volume of 10 μl with 30 min incubation at room temperature. An upstream region from Arabidopsis PP2A (-1/-150) and an upstream region covering the AW-box from BCCP2 (-2/-152) was used as negative and positive control, respectively. Target probes for AtWRI1 upstream regions consisted of 150 bp fragments. Sequences for primers can be found in Supplementary Table S1. The DNA–protein complexes were resolved on 10 % non-denaturing polyacrylamide gels and visualized using ChemiDoc^TM^ MP Imaging System (Bio-Rad Laboratories, Hercules, CA, United States). Gel pictures have been trimmed, resized, and brightness and contrast have been changed on the raw format files using Adobe Photoshop CC (Adobe Systems, San Jose, CA, United States). All gel pictures have been treated identically.

## Results

### Verification of *N. benthamiana* Transactivation System for Promotor Activity Assay

The potential use of an *N. benthamiana* transient expression system for transactivation studies on Arabidopsis promoter elements was verified using a 2000 bp upstream region from Arabidopsis (*AtWRI1up2000*) that was cloned into a binary vector carrying a GUS-reporter gene. To verify proper vector functionality, a CaMV 35S promoter and the coding sequence from Arabidopsis *ABSCISIC ACID INSENSITIVE 3* (*AtABI3*) were also cloned into the same vector as upstream elements for a positive and negative control, respectively. All constructs were separately agroinfiltrated into *N. benthamiana* leaves and the GUS expression was visualized after 4 days. Both the *35S::GUS* and *AtWRI1up2000::GUS* showed high GUS expression while the *ABI3::GUS* showed no signs of GUS expression at all (Supplementary Figure S1). To test whether competition for general TFs between transiently expressed promoters would influence the system, a gradient of *35S::GFP* was used. This showed that no negative effect of increasing expression of GFP on *AtWRI1up2000* transcription efficiency could be detected (Figure 2), showing that the observed results were not due to a depletion of general TFs or response to increased infection by *A. tumefaciens*.

### Arabidopsis WRI1 Upstream Region Is Transcriptionally Active in *N. benthamiana* Leaves

**FIGURE 1:**
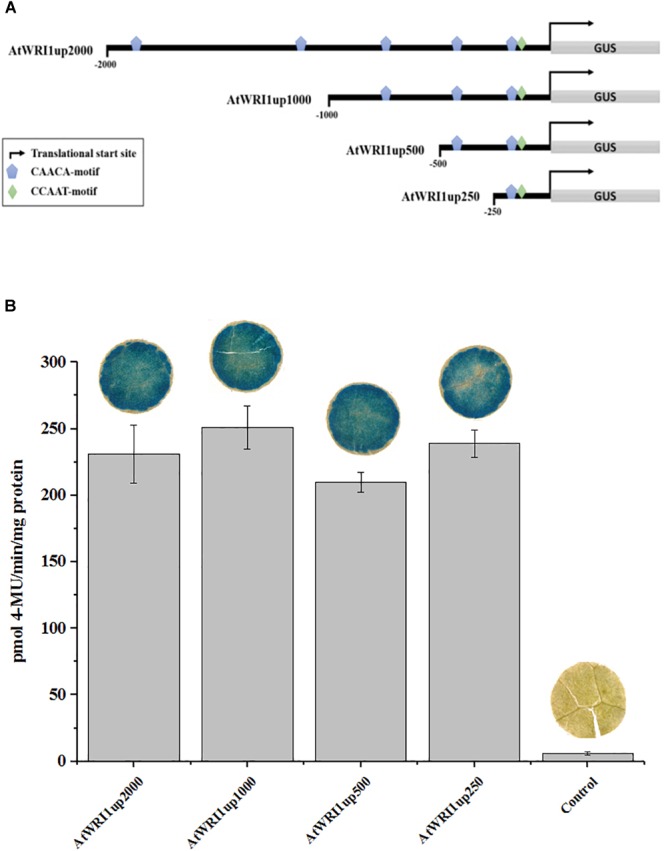
Characterization of transcriptional functionality of AtWRI1 upstream regions during transient in *N. benthamiana* leaf. **(A)** Simplified architecture of the AtWRI1 upstream regions used in this study fused with the GUS reporter gene. **(B)** Absolute quantification of GUS activity in *N. benthamiana* leaf tissue infiltrated with respective AtWRI1up::GUS construct (OD_AtWRI1up_ = 0.6). Control shows leaf tissue with 35S::GFP (OD_35S:GFP_ = 0.6). Bars show an average between three biological samples. Pictures of GUS activity visualization show a representative leaf disc. Error bars = SD.

Following the observation that the *AtWRI1up* 2000 bp region was transcriptionally active, 1000, 500, and 250 bp genomic regions upstream of *AtWRI1* ATG start codon was isolated and cloned in front of the *GUS* reporter gene. *In silico* motif analysis failed to identify a credible TATA-box but was able to identify a CCAAT-box close to the predicted transcription start site (Figure 1A). CCAAT is known to be a target sequence for LEAFY COTYLEDON1 (Lotan et al., 1998) which in turn has been shown to regulate fatty acid biosynthesis through AtWRI1 (Mu et al., 2008). Furthermore, a total of five CAACA-motifs were identified within the 2000 bp upstream region (Figure 1A). The CAACA-motif has been identified as the target sequence for the AP2-domain of the RELATED TO ABI3/VP1-1 TF (Kagaya et al., 1999). No AW-box could be identified within the 2000 bp region. Analysis of absolute promoter strength through GUS-quantification was carried out by transient expression in *N. benthamiana* leaves and showed that all upstream regions were similarly able to induce transcription of the GUS-reporter gene (Figure 1B).

### WRI1 Expression Is Autoregulated Through a Conserved Mechanism

**FIGURE 2:**
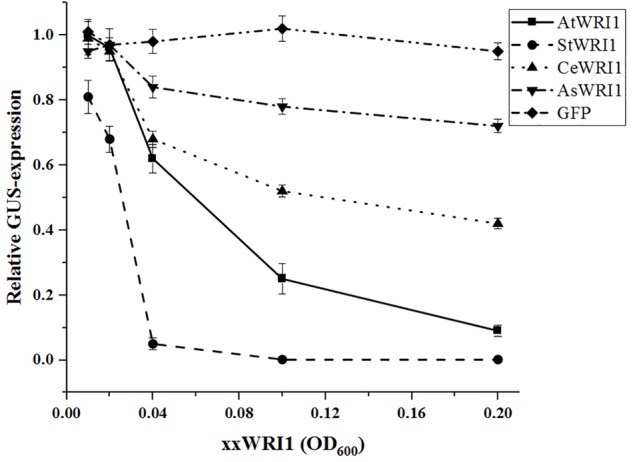
Relative quantification of transcriptional activity of AtWRI1up2000::GUS when co-infiltrated into *N. benthamiana* leaves with increasing OD of WRI1 homologs. AtWRI1up2000::GUS was maintained at OD = 0.6 while 35S::xxWRI1 OD gradually increased. Normalization was done against an average from a triplicate of AtWRI1up2000::GUS (OD = 0.6). Points show the average of three biological samples. Error bars = SD.

To explore if *WRI1* could be under transcriptional autoregulation the Arabidopsis 2000 bp upstream region cloned into the GUS-reporter gene containing vector was co-infiltrated into *N. benthamiana* leaves together with vectors carrying *WRI1* homologs from Arabidopsis, potato, yellow nutsedge, and oat (*AtWRI1, StWRI1, CeWRI1*, and *AsWRI1*, respectively) behind the 35S-promoter. To simulate variations in *WRI1* expression different OD_600_ of Agrobacteria containing *WRI1*-vectors was used. A recent study has confirmed that increasing culture density of infiltrated Agrobacterium leads to increased GUS expression in *N. benthamiana* leaves (Norkunas et al., 2018). As shown in Figure 2, increasing expression of all four WRI1 homologs strongly affects the transcriptional activity of the Arabidopsis upstream region negatively, indicating the presence of an evolutionary conserved autoregulatory function of WRI1. In support for this, AtWRI1 has a more severe effect on its native upstream region while the autoregulatory effect becomes weaker with lower sequence similarities of *WRI1* from other species (Supplementary Figure S2) with AsWRI1 showing the weakest autoregulation on the Arabidopsis WRI1 upstream region. Interestingly, we observed that the StWRI1 is drastically more effective in downregulating the activity of the Arabidopsis upstream region than the AtWRI1 possibly reflecting the more close relatedness between *N. benthamiana* and potato (Figure 2).

### The Target Sequence for Autoregulation Is Situated Close to the Translation Start Site

**FIGURE 3:**
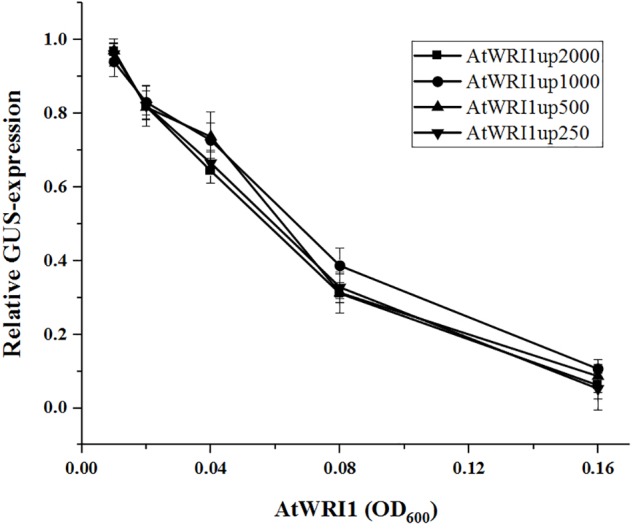
Relative quantification of truncated AtWRI1 upstream region transcriptional activity when co-infiltrated in *N. benthamiana* leaves with 35S::AtWRI1 at different OD. Infiltration OD of all AtWRI1up truncations were kept at 0.6 for all treatments. Normalization was done against an average from a triplicate of AtWRI1up2000::GUS (OD = 0.6). Points show the average of three biological samples. Error bars = SD.

In an effort to identify the target sequence of importance for the autoregulatory mechanism of WRI1, we generated gene constructs with truncated versions of the initial 2000 bp long Arabidopsis upstream region (Figure 1A) behind the GUS-reporter gene which were co-infiltrated together with a gradient of vectors enabling constitutive expression of *AtWRI1* into *N. benthamiana* leaves. Interestingly, all truncated variations of *AtWRI1up2000* showed very similar capacity for transcription initiation as the full-length *AtWRI1up2000* (Figure 3). All upstream length variants also showed a very similar response to increased AtWRI1 as the full-length region (Figure 3) showing that the minimal promoter required for both basal expression and autoregulatory function is shorter than 250 bp.

### WRI1 Has Three Evolutionarily Conserved Intrinsically Disordered Regions

WRI1 consists of two highly conserved AP2-domains, separated by a highly conserved 30 amino acid long spacer and flanked by two highly variable regions, the shorter N-terminus adjacent region and the longer C-terminus adjacent region (Ma et al., 2013; Grimberg et al., 2015). For AtWRI1, both flanking regions have been shown to mainly be made up of IDRs where the IDR adjacent to the C-terminus is important for the AtWRI1 turnover rate through the presence of a PEST-motif situated therein (Ma et al., 2015). The C-terminal PEST-motif was also present in CeWRI1, and AsWRI1 but interestingly not in StWRI1. In addition to this, the PEST-motif present in IDR1, close to the n-terminus, previously described by Ma et al. (2015) in AtWRI1 was also shown to be present in AsWRI1 (Supplementary Figure S3).

**FIGURE 4:**
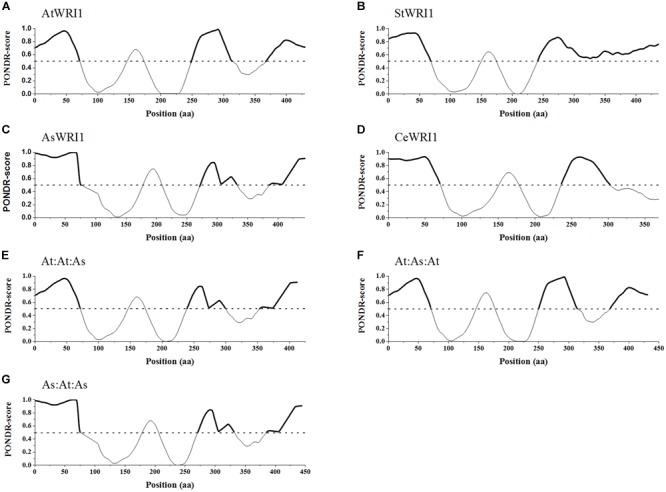
Predicted intrinsically disordered regions within native WRI1 homologs and recombinant WRI1 permutations. **(A–D)** show naturally occurring WRI1 homologs from four plant species. **(A)** AtWRI1; Arabidopsis WRI1, **(B)** StWRI1; potato WRI1, **(C)** AsWRI1; oat WRI1, and **(D)** CeWRI1; yellow nutsedge WRI1. **(E–G)** show the recombinant permutations of AtWRI1 and AsWRI1. **(E)** AtWRI1_n_:At:WRI1_AP2_:AsWRI1_c_, **(F)** AtWRI1_n_:As:WRI1_AP2_:AtWRI1_c_, and **(G)** AsWRI1_n_: At:WRI1_AP2_:AtWRI1_c_. All predictions were done using PONDR VL3-BA algorithm. Predicted intrinsically disordered regions marked by bold lines. Dashed line marks a PONDR-score of 0.5.

To see if the three IDR regions identified in AtWRI1 were conserved in the three additional homologs *in silico* prediction of disordered regions were run using the VL3-BA algorithm (Figure 4A–D). This analysis revealed that although there is no homology of the amino acid sequence within the AP2-domain flanking regions, the presence of IDRs is structurally very well conserved. When comparing with AtWRI1, all homologs share the first and second IDR with only AsWRI1 deviating due to an insertion creating a spacer between the IDR and first AP-domain (Figure 4C). The third IDR is present in at least AtWRI1 and AsWRI1 and appear to be present but fused with the second IDR in StWRI1 (Figure 4B). The missing IDR3 in CeWRI1 is likely due to a large deletion in the C-terminus of CeWRI1 where approximately 55 amino acids have been lost. The fact that this deletion not has affected the transactivation activity (Grimberg et al., 2015) or the ability to autoregulate the Arabidopsis upstream region (Figure 2) of CeWRI1 indicate that IDR3 is likely not necessary for the autoregulatory mechanism to function.

### The Presence of AsWRI1 Regions in AtWRI1 Increases Oil Accumulation in *N. benthamiana* Leaf Tissue

**FIGURE 5:**
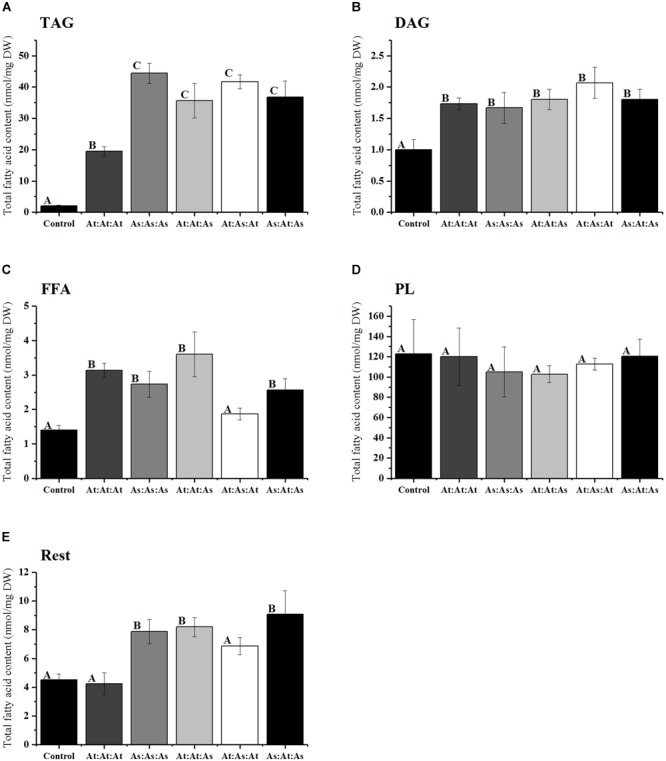
Fatty acid analysis of different lipid classes (nanomoles per milligram dry weight) from infiltrated *N. benthamiana* leaf tissue infiltrated with five different constructs of WRI1 permutations consisting of the WRI1 N-region, AP2-region, and C-region, respectively, and where these domains originated from either AtWRI1 or AsWRI1. **(A)** TAG; triacylglyceride, **(B)** DAG; diacylglycerol, **(C)** FFA; free fatty acids, **(D)** PL; polar lipids, and **(E)** Rest fraction. Bars represent average from three biological replicates. Error bars = SD. Letters indicate significantly distinguishable groups based on Tukey’s test with cutoff at *P* ≤ 0.05.

Based on these observations, we concluded that WRI1 can be divided into three distinct regions; (i) N-region, (ii) AP2-region, and (iii) C-region (Supplementary Figure S2). The N-region was defined as stretching from the N-terminus over the first IDR up until the first conserved amino acid of the first AP2-domain (Figure 4E–G). The AP2-domain consists of the two AP2-domains stretching from the first conserved amino acid of the first AP2-domain until the last conserved amino acid of the second AP2-domain. To evaluate the role of these three regions in the autoregulation of Arabidopsis *WRI1* upstream region, we made three different constructs by swapping domains between AtWRI1 and AsWRI1. The resulting WRI1 permutations were cloned behind a 35S-promoter and their functionality was evaluated by agroinfiltration into *N. benthamiana* leaves which were analyzed for content of different lipids (Figure 5). The increase in TAG content showed that all permutations were fully functional and capable of the transactivation of downstream WRI1-targets involved in fatty acid biosynthesis. Surprisingly, we observed that all incorporations of regions from AsWRI1 significantly increased the TAG-content compared to native AtWRI1. This is probably due to either an increase in WRI1 stability, an increased transactivation activity, or both. At least the C-terminus of AtWRI1 is known to be important for proper transactivation activity (Masaki et al., 2005).

### The Two Conserved AP2-Domains inWRI1 Are Important for Autoregulation

**FIGURE 6:**
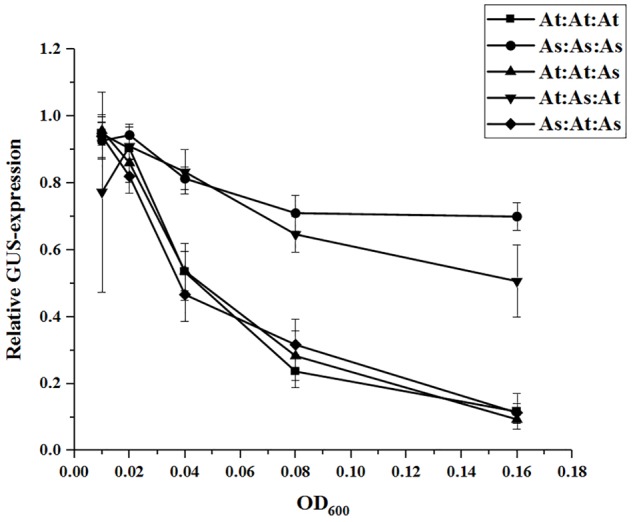
Relative quantification of AtWRI1up2000 transcriptional activity when co-infiltrated in *N. benthamiana* leaves with WRI1-permutations at different OD. Infiltration OD of AtWRI1up2000::GUS were kept at 0.6 for all treatments. Normalization was done against an average from a triplicate of AtWRI1up2000::GUS (OD = 0.6). Points show the average of three biological samples. Error bars = SD.

Next, we co-infiltrated constructs with the WRI1 permutations (behind CaMV 35S promoters), in the same manner as previously, together with the *AtWRI1up2000::GUS* construct and the promoter activity was analyzed through GUS-activity assays (Figure 6). Increased expression of the permutations At:At:As and As:At:As was as efficient in quenching the *AtWRI1up2000* promoter activity as the native AtWRI1 while At:As:At exerted a similar influence as the native AsWRI1, indicating that WRI1 autoregulation is closely connected to the two evolutionary conserved AP2-domains.

### WRI1 Is Not Able to Interact With Its Own Upstream Region

**FIGURE 7:**
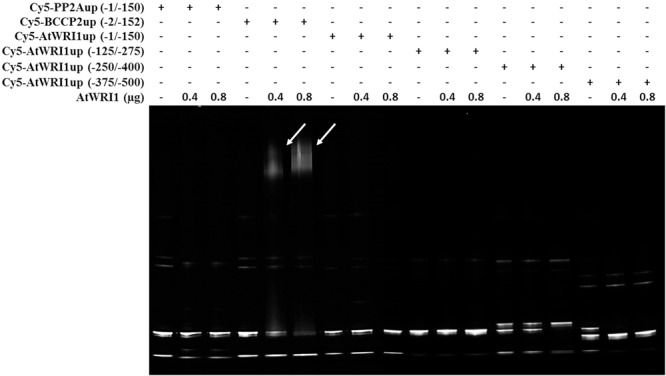
Fluorescent EMSA using Cy5-labeled DNA-fragments and purified decahistidine tagged Arabidopsis WRI1. Upstream regions from PP2A (lane 1, 2, and 3) and BCCP2 (lane 4, 5, and 6) was used as negative and positive control, respectively. Poly dI-dC was used as competitor. Arrows indicate shifted band.

To evaluate the ability of AtWRI1 to interact directly with its upstream region decahistidine tagged AtWRI1 was expressed in *E. coli* and purified for binding studies. Potential DNA–protein interactions were analyzed using fEMSA with overlapping 150 bp long fragments from the *AtWRI1up250*-region. A 150 bp fragment from the Arabidopsis upstream region of *PP2A* was used as a negative control as it appears to be under no regulation from WRI1 (Grimberg et al., 2015) and an *in silico* analysis revealed no AW-box. The 150 bp fragment from the Arabidopsis upstream region of *BCCP2* used as a positive control contains an AW-box and is an experimentally confirmed target of *AtWRI1* (Baud et al., 2009; Maeo et al., 2009). The fEMSA clearly show that AtWRI1 is not capable of independently interacting with the first 500 bp of its own upstream region. It is, however, able to interact with the AW-box from the BCCP2 upstream region, used here as a positive control, under identical conditions (Figure 7).

## Discussion

During the seed maturation process, carbon moieties are generally stored either as starch or TAG to be utilized as energy during germination. The seed maturation, with accumulation of storage compounds, is regulated through large and complex changes in the developmental program. The evolutionary conserved TF WRI1 has been shown to play a central role in this process by redirecting carbon flow from starch synthesis to de novo fatty acid synthesis (Focks and Benning, 1998; Cernac and Benning, 2004; Grimberg et al., 2015). In nature WRI1 mainly functions within a seed developmental context but has been shown to not require a seed environment to induce oil accumulation in both other sink tissue as well as in source tissue (Vanhercke et al., 2014; Hofvander et al., 2016). Despite the importance of WRI1 in oil seed development and the great interest to use *WRI1* in biotechnological applications, most research efforts have been focused on either the downstream effect of WRI1 or placing WRI1 in a larger developmental program including other TFs. Up until recently, limited research has been focused on understanding the internal details of the WRI1 regulatory system. In this study, we investigate this topic and show that WRI1 is subject to an evolutionary conserved negative autoregulation.

It is known that mRNA levels of *WRI1* in mature Arabidopsis leaf tissue are very low, close to non-detectable (Schmid et al., 2005). Similar observations have been made for *N. benthamiana* leaf tissue where *WRI1* mRNA is present only at very low level (Grimberg et al., 2015). These observations fit well with the previously proposed model where the roles of WRI1, WRI3, and WRI4 mainly are determined by differential expression patterns based, at least in part, on promotor control (To et al., 2012). Interestingly, our study shows that transiently introduced *AtWRI1* upstream region is highly active in initiating transcription in mature *N. benthamiana* leaf tissue. Previous *WRI1* promotor activity studies have mainly been conducted through stable transformation of Arabidopsis and were focused on early developmental stages and seed development where the *WRI1* promoter was shown to be active in embryonic tissue but not in mature leaf tissue (Baud et al., 2007; Wang et al., 2016). Promotor activity of the maize *WRI1* promoter in BMS cell culture also indicated very low baseline activity (Shen et al., 2010). Our observations raise the question of why transiently introduced *WRI1* upstream regions are capable to effectively initiate transcription while similar constructs in stable transformants remain silent. The lack of chromatin structure in the introduced plasmid is one likely explanation, making chromatin-based epigenetic silencing of the region impossible. This study was not designed to investigate why the transiently introduced *WRI1* upstream region is able to initiate transcription. But the reductionist properties of this system offer the capacity for high-resolution interaction studies between *WRI1* regulatory elements and TFs in a manner otherwise not possible.

In this study, we have shown that the ability of the Arabidopsis upstream region of *WRI1* to initiate transcription *in planta* is negatively affected by the presence of WRI1. This confirms the observation made by Cernac and Benning (2004) that the Arabidopsis *wri1-1* mutant show increased expression of the mutant gene in the developing seed. In addition, we also show that the autoregulatory mechanism is evolutionarily conserved between dicotyledons and monocotyledons and thus most likely was present in the last common ancestor. The high conservation level of this autoregulatory mechanism is not surprising when taking into account that WRI1 itself is to a large extent evolutionary conserved, both in structure and in function (Grimberg et al., 2015). Despite this, it is clear that different WRI1 homologs assert different levels of influence on the *AtWRI1* upstream region. A previous study of WRI1 isolated from rapeseed proposes that the BnWRI1s ability to induce oil accumulation when expressed in an Arabidopsis seed environment is due to BnWRI1 not interfering with endogenous WRI1 expression (Liu et al., 2010). Based on our findings, this is likely not the case but a more probable explanation would be that BnWRI1 interferes with the regulatory elements of *WRI1* to a lower degree than the native WRI1 does.

It has previously been reported that AtWRI1 contains three IDRs, one close to the N-terminus and two toward the C-terminus (Ma et al., 2015). We could show that the two IDRs flanking the double AP2-domain are conserved throughout the four analyzed WRI1 homologs from Arabidopsis, potato, yellow nutsedge, and oat. The third IDR, which is located toward the C-terminus, is not present in CeWRI1 and appears to have merged with the second IDR in StWRI1. While removal of IDR2 and IDR3 from AtWRI1 have been shown to eliminate its transactivation capability, removing only IDR3 resulted in increased protein stability due to the removal of a PEST motif situated within (Masaki et al., 2005; Ma et al., 2015). PEST motifs have been shown to act as signals for proteolytic activity and to be associated with IDRs (Rogers et al., 1986; Rechsteiner and Rogers, 1996; Singh et al., 2006). For the here studied WRI1 homologs, the predicted IDR3 PEST motif was found present in AtWRI1 and AsWRI1 but not in StWRI1 or CeWRI1. This indicates that the instability of WRI1 through the presence of a PEST motif does probably not directly interfere with its autoregulatory mechanism.

The swapping of the IDR-containing regions between AsWRI1 and AtWRI1 showed clearly that the autoregulatory mechanism is entirely reliant upon the double AP2-domains. Since no known AW-box could be identified in the upstream region of AtWRI1, this strongly indicates that the autoregulatory mechanism is indirect and dependent on downstream target genes of WRI1. This is also supported by the fEMSA results showing the inability of AtWRI1 to independently bind to the first 500 bp of its own upstream region. In conclusion, this shows that the autoregulatory mechanism either is a result of downstream actions of WRI1 or as a response to changes in the metabolome.

Furthermore, it appears as if the variation observed between different WRI1 homologs in their ability to autoregulate the AtWRI1 upstream region correlates well with their evolutionary distance to *N. benthamiana* (Sarkinen et al., 2013; The Angiosperm Phylogeny Group et al., 2016). The closest relative to *N. benthamiana* in our study is potato (Sarkinen et al., 2013) and the corresponding StWRI1 also has the most severe decrease in the transcriptional rate of the introduced upstream region. The most remote relative to *N. benthamiana* in our study is oat (The Angiosperm Phylogeny Group et al., 2016) and AsWRI1 is also the least effective in decreasing the expression of the Arabidopsis upstream region in the *N. benthamiana* system. The same is also true for the WRI1 homologs from Arabidopsis and yellow nutsedge which are the second and third closest relatives of *N. benthamiana*, respectively (The Angiosperm Phylogeny Group et al., 2016). A possible explanation to this observation is that the WRI1 homologs stemming from species that are closely related to *N. benthamiana*, such as potato from the same family (Solanaceae), is better at activating genes outside the network of traditional core genes targeted by WRI1. This explanatory model is to some degree supported by the observation that although AsWRI1 influences fewer genes in *N. benthamiana* than StWRI1 it is still able to increase the oil accumulation to a larger degree than StWRI1 (Grimberg et al., 2015). This would mean that the protein responsible for the observed autoregulatory effect of WRI1 likely is transcribed by a gene not belonging to the described network of core target genes of WRI1 but under a still evolutionary conserved trans-regulatory interaction.

Based on the results presented in this study, a more complex picture of the regulatory system governing the expression and activity of WRI1 emerges where increased WRI1 expression decreases the transcriptional activity of its upstream region. This raises the question of the biological purpose of this system. In addition to constituting a “fail–safe” to inhibit expression of WRI1, and the resulting accumulation of fatty acids and TAG, when not required, the temporal variation of photosynthate levels in source tissue could hold a key to the answer. In plant leaves, starch is being accumulated during daytime and remobilized to sucrose during non-photosynthetic periods in a diurnal pattern (Geiger and Servaites, 1994; Geigenberger and Stitt, 2000). Similar patterns have been observed for genes involved in lipid biosynthesis (Hsiao et al., 2014; Nakamura et al., 2014) as well as indications that AtWRI1 expression follows a similar diurnal cycle (Mockler et al., 2007) raising the question whether the autoregulatory system of WRI1 has evolved to allow for a diurnal expression pattern warranting further research into WRI1 temporal expression patterns.

The potential of using *WRI1* in biotechnological applications to, e.g., increase oil biosynthesis in already oil accumulating tissues or to reprogram traditional starch and sugar accumulating source tissues into oil accumulating sink tissues is well known and has been discussed in depth before (Shih et al., 2016; Kang et al., 2017; Wan et al., 2017). With the technological advances in recent years and the increased knowledge of gene regulation, we can now utilize transcriptional regulators more predictably. There has also been a great interest within synthetic biology to use artificial TFs to regulate specific genes (Liu and Stewart, 2015; Nielsen and Keasling, 2016). In this work, we have shown that it is possible through domain swapping between naturally occurring homologs to create a semi-synthetic WRI1 that is able to maintain the transactivation capacity of the parent homologs. In addition to this, it was also possible with these semi-synthetic WRI1 to change the autoregulatory feedback loop, thereby probably less severely influencing the native WRI1/promotor interaction. This enables the establishment of an autoregulatory feedback loop with little interference on naturally occurring developmental programs. It would also be possible to exchange specific domains of native *WRI1* with other *WRI1* homologs through the use of CRISPR/Cas9-dependent technology tailoring semi-synthetic genes *in planta* without the need for traditional biotechnological tools. Further studies into the promotor architecture of *WRI1* will allow for deeper understanding of this autoregulatory mechanism allowing for more detailed and tailored application of this mechanism in other approaches.

## Data Availability

All datasets generated for this study are included in the manuscript and/or the Supplementary Files.

## Author Contributions

PH and AC envisioned this study and coordinated the experimental work. PH, ÅG, and PS designed the experimental setup. PS drafted the manuscript. PH, AC, and ÅG contributed to the final version. PS performed the experimental work.

## Conflict of Interest Statement

The authors declare that the research was conducted in the absence of any commercial or financial relationships that could be construed as a potential conflict of interest.

## Footnotes

1 http://www.pondr.com/

2 http://emboss.bioinformatics.nl/cgi-bin/emboss/epestfind
