# Supplementary material for: WRINKLED1 Is Subject to Evolutionary Conserved Negative Autoregulation
Source: Front Plant Sci. 2019 Mar 28;10:387. doi: 10.3389/fpls.2019.00387 (PMC6447653; doi:10.3389/fpls.2019.00387)
Supplement: Supplementary file 1 [file Data_Sheet_1.docx]

Supplementary Material

WRINKLED1 is subject to evolutionary conserved negative autoregulation

Per Snell^*^, Åsa Grimberg, Anders S. Carlsson and Per Hofvander

*** Correspondence:** Per Snell, [per.snell@slu.se](mailto:per.snell@slu.se)


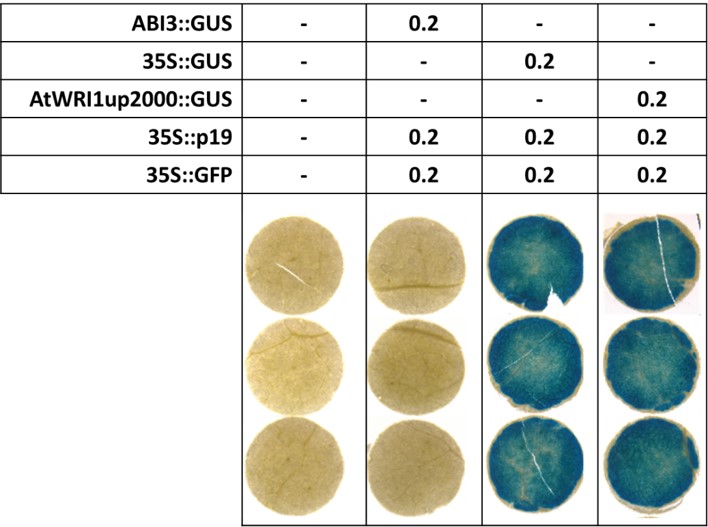


**Figure S1**. Uninfiltrated (A) and infiltrated (B-D) reference leaf discs. Table show final OD_600_ in infiltration solution for *A. tumefaciens* carrying respective expression plasmid construct. Pictures show GUS-expression in leaf discs (Ø 8 mm) in triplicate.


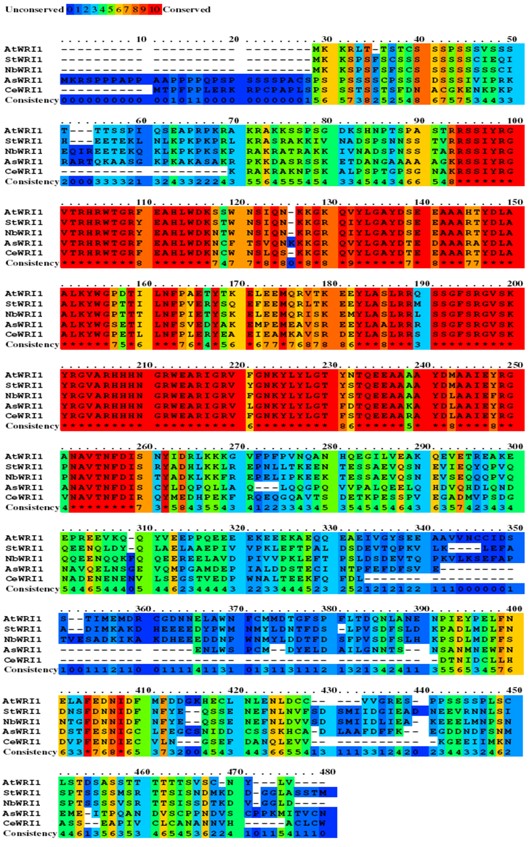


**Figure S2.** Sequence alignment of five WRI1 homologs based on amino acid conservation. The two AP2 domains together with the conserved spacer region have been underlined with black. The N-region is underlined in green while the C-region use red.


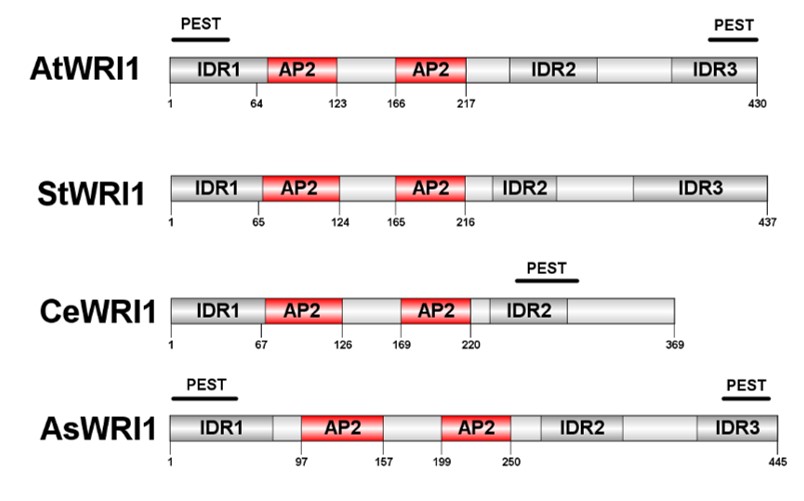


Figure S3. Predicted PEST motifs identified in four (arabidopsis (At), potato (St), yellow nutsedge (Ce) and oat (As)) WRI1 homologs. PEST motifs were identified using ePESTfind (<http://emboss.bioinformatics.nl/cgi-bin/emboss/epestfind>) and the standard threshold score of +5.0.

Table S1. Table over primers used in this study.

| *Gene/region* | *Primer name* | *Primer sequence* | *Note* |
| --- | --- | --- | --- |
| *Primers used for AtWRI1 upstream region isolation and truncation* | | | |
| *AtWRI1up2000* | *attB-AtWRI1up2000_for* |  |  |
|  | *attB-AtWRI1up2000_rev* |  |  |
| *AtWRI1up1000* | *attB-AtWRI1up1000_for* | GGGGACAAGTTTGTACAAAAAAGCAGGCTAAATGATAACAGTTAGAACATCCAA | attB-AtWRI1up2000_rev used as reverse primer |
| *AtWRI1up500* | *attB-AtWRI1up500_for* | GGGGACAAGTTTGTACAAAAAAGCAGGCTGGCTACTATTTTTCTTTTCTAAAAC | attB-AtWRI1up2000_rev used as reverse primer |
| *AtWRI1up250* | *attB-AtWRI1up250_for* | GGGGACAAGTTTGTACAAAAAAGCAGGCTAAAAAAGAAAGACAGCGTGGAG | attB-AtWRI1up2000_rev used as reverse primer |
| *Primers used for WRI1 permutations* | | | |
| *AtWRI1* | *attB-AtWRI1_for* | GGGGACAAGTTTGTACAAAAAAGCAGGCTATGAAGAAGCGCTTAACCACTTCC |  |
|  | *attB-AtWRI1_rev* | GGGGACCACTTTGTACAAGAAAGCTGGGTTCAGACCAAATAGTTACAAGAAACCGA |  |
| *AsWRI1* | *attB-AsWRI1_for* | GGGGACAAGTTTGTACAAAAAAGCAGGCTATGAAGAGATCCCCGCCTCC |  |
|  | *attB-AsWRI1_rev* | GGGGACCACTTTGTACAAGAAAGCTGGGTTCAATTACACACAGTGATCA |  |
| *AtWRI1_N_:AsWRI1_AP2_* | *At_N_:As_AP2__rev* | CAAGCCCTGCTTCTACCCGAAGGAGTTCCATCTACAGGGG | attB-AtWRI1_for used as forward primer |
| *AsWRI1_N_:AtWRI1_AP2_* | *As_N_:At_AP2__rev* | GCCGCCGCCGGGAAGCGCAGCTCTATCTACAGAGG | attB-AsWRI1_for used as forward primer |
| *AsWRI1_N_:AtWRI1_AP2_:AsWRI1_C_* | *AsWRI1_N_:AtWRI1_AP2__for* | CCTCTGTAGATAGAGCTGCGCTTCCCGGCGGCGGC |  |
|  | *AtWRI1_AP2_:AsWRI1_AP2__rev* | AACGCGGTTACTAATTTCGACATTAGTTGCTACCTGGACCAGCCACA |  |
| *AtWRI1_N_:AsWRI1_AP2_:AtWRI1_C_* | *AtWRI1_N_:AsWRI1_AP2__for* | CCCCTGTAGATGGAACTCCTTCGGGTAGAAGCAGGGCTTG |  |
|  | *AsWRI1_AP2_:AtWRI1_AP2__rev* | GGTTACTAATTTCGACATTAGTTGCTACCTGGACCAGCCAC |  |
| *AtWRI1_AP2_:AsWRI1_C_* | *AtWRI1_AP2_:AsWRI1_C__for* | TGTGGCTGGTCCAGGTAGCAACTAATGTCGAAATTAGTAACCGCGTT | attB-AtWRI1_rev used as reverse primer |
| *AsWRI1_AP2_:AtWRI1_C_* | *AsWRI1_AP2_:AtWRI1_C__for* | GTGGCTGGTCCAGGTAGCAACTAATGTCGAAATTAGTAACC | attB-AsWRI1_rev used as reverse primer |
| *Primers used for EMSA analysis* | | | |
| *AtWRI1 without start codon* | *attB-AtWRI1(-sc)_for* | GGGGACAAGTTTACAAAAAAGCAGGCTTGAAGAAGCGCTTAACCACTTCCA |  |
|  | *attB-AtWRI1_rev* | GGGGACCACTTTGTACAAGAAAGCTGGGTTCAGACCAAATAGTTACAAGAAACCGA |  |
| *PP2Aup (-1/-150)* | *PP2Aup_(-1/-150)_for* | GGATTTAGAAGAAGAGAAATTGTGAATTT | Cy5-labeled |
|  | *PP2Aup_(-1/-150)_rev* | AAAATGAAAGCCACGTGTCAGAC | Cy5-labeled |
| *BCCP2up (-2/-151)* | *BCCP2up_(-2/-151)_for* | TAAAAGAGTTGGGTTTCTTCGTAAG | Cy5-labeled |
|  | *BCCP2up_(-2/-151)_rev* | TTGAGACAGTGGACGATGAAACC | Cy5-labeled |
| *AtWRI1up (-1/-150)* | *AtWRI1up_(-1/-150)_for* | AATTAACCCAATTAGCCCTTTCCTC | Cy5-labeled |
|  | *AtWRI1up_(-1/-150)_rev* | ACTCTGAGAAAGTTTAGATTTTTTTTTGGA | Cy5-labeled |
| *AtWRI1up (-125/-275)* | *AtWRI1up_(-125/-275)_for* | TAAAAAAAAATACAAAAATACAAAGAAAAAAGAAAG | Cy5-labeled |
|  | *AtWRI1up (-125/-275)_rev* | GAGGAAAGGGCTAATTGGGTTAATT | Cy5-labeled |
| *AtWRI1up (-250/-400)* | *AtWRI1up (-250/-400)_for* | GGCTACTATTTTTCTTTTCTAAAACCAAA | Cy5-labeled |
|  | *AtWRI1up (-250/-400)_rev* | AAAATATCTTAATTTGTTTTTTTGCAATTCTAT | Cy5-labeled |
| *AtWRI1up (-375/-500)* | *AtWRI1up (-375/-500)_for* | GCAAAAAAACAAATTAAGATATTTTACATTCC | Cy5-labeled |
|  | *AtWRI1up (-375/-500)_rev* | CTTTGTATTTTTGTATTTTTTTTTACGTTTTTTTA | Cy5-labeled |

**Table S2.** Table over protein sequences used in this study.

| **Name** | **Sequence** |
| --- | --- |
| AtWRI1 | MKKRLTTSTCSSSPSSSVSSSTTTSSPIQSEAPRPKRAKRAKKSSPSGDKSHNPTSPASTRRSSIYRGVTRHRWTGRFEAHLWDKSSWNSIQNKKGKQVYLGAYDSEEAAAHTYDLAALKYWGPDTILNFPAETYTKELEEMQRVTKEEYLASLRRQSSGFSRGVSKYRGVARHHHNGRWEARIGRVFGNKYLYLGTYNTQEEAAAAYDMAAIEYRGANAVTNFDISNYIDRLKKKGVFPFPVNQANHQEGILVEAKQEVETREAKEEPREEVKQQYVEEPPQEEEEKEEEKAEQQEAEIVGYSEEAAVVNCCIDSSTIMEMDRCGDNNELAWNFCMMDTGFSPFLTDQNLANENPIEYPELFNELAFEDNIDFMFDDGKHECLNLENLDCCVVGRESPPSSSSPLSCLSTDSASSTTTTTTSVSCNYLV |
| AsWRI1 | MKRSPPPAPPAAPPPPQPSPSSSSPACSPSPSSSSCPSSSDSSSIVIPRKRARTQKAASGKPKAKASAKRPKKDASRSSKETDANGAAAAAGKRSSIYRGVTRHRWTGRFEAHLWDKNCFTSVQNKKKGRQVYLGAYDTEDAAARAYDLAALKYWGSETILNFSVEDYAKEMPEMEAVSREEYLAALRRRSSGFSRGVSKYRGVARHHHNGRWEARIGRVLGNKYLYLGTFDTQEEAAKAYDLAAIEYRGANAVTNFDISCYLDQPQLLAQLQQGPQVVPALQEELQHDVQHDLQNDNAVQELNSGEVQMPGAMDEPIALDDSTECINTPFEFDFSVEENLWSPCMDYELDAILGNNTSNSANMNEWFNDSTFESNIGCLFEGCSNIDDCSSSKHCADLAAFDFFKEGDDNDFSNMEMEITPQANDVSCPPNDVSCPPKMITVCN |
| StWRI1 | MKKSPSFSCSSSSSSSSCIEQIHEETEKLNLKPKPKPRLKRASRAKKIVNADSPSNNSSTVRRSSIYRGVTRHRWTGRYEAHLWDKSTWNSIQNKKGRQIYLGAYDSEEAAARTYDLAALKYWGPTTILNFPVERYSQEFEEMQRLTKEEYLASLRRMSSGFSRGVSKYRGVARHHHNGRWEARIGRVYGNKYLYLGTYSTQEEAAAAYDMAAIEYRGPNAVTNFDISRYADHLKKLREPNLLTKEENTESSAEVQSNEVIEQYQPVQQEENQLDYQLAELAAEPIVVPKLEFTPALDSDEVTQPKVLKLEFAADIMKAKDHEEEEDYPWMNMYLDNTFDSLPVSDFSLDKPADLMDLFNDNSFDNNIDFNFYEQSSENEFNLNVFSDSMIIDGIEADNEEVRNNLSISPTSSSSMSRTTSISNDMKDDGGLASSTM |
| NbWRI1 | MKKSPSFSFSCSSSSSSSSSCIEQIREETEKQKLKPKPKSKPRAKRATRAKKIVNADSPNSSTARRSSIYRGVTRHRWTGRYEAHLWDKNTWNSIQNKRGRQIYLGAYDSEEAAARTYDLAALKYWGPTTTLNFPIETYSKELEEMQRISKEMYLASLRRLSSGFSRGVSKYRGVARHHHNGRWEARIGRVFGNKYLYLGTYSTQEEAAAAYDLAAIEFRGPNAVTNFDISTYADKLKKFREPELIPKEEKTESSAEVQSNEVSEQQQPVQQQEENQQKFQQEEREELAVDPIVVPKLEFTPSLDSDEVTQPKVLKSEFAPTVESADKIKAKDHEEEDDNPWNMYLDDTFDSFPVSDFSLHKPSDLMDFFSNTGFDNNIDFNFYEQSSENEFNLDVVSDSMIIDLIEAKEEELMNPSNSPTSSSSVSRTTSISSDTKDVGGLD |
| CeWRI1 | MTPFPPLERKRPCPAPLSPSSSTSSTSFDNACGKENKPKPKRAKRAKNPSKALPSPTGPSGNAKRSSIYRGVTRHRWTGRFEAHLWDKNCWNSLQSKKGKQVYLGAYDTEEAAARTYDLAALKYWGPETLLNFPLERYEAEIEAMKAVSRDEYLASLRRSSSGFSRGVSKYRGVARHHHNGRWEARIGRVFGNKYLYLGTFSTQEEAARAYDLAAIEYRGVNAVTNFDIRQYMEDHPEKFRQEQGQAVTSDETKPESSPVEGADMVPSDGNADENENENVLSEGSTVEDPWNALTEEKFQFDLDTNIDCLLHDVPFENDIECVLNGSEFDANQLEVVKGEEIIMKNASSEAPIVCLCANANNVHACLCW |
| As:At:As | MKRSPPPAPPAAPPPPQPSPSSSSPACSPSPSSSSCPSSSDSSSIVIPRKRARTQKAASGKPKAKASAKRPKKDASRSSKETDANGAAAAAGKRSSIYRGVTRHRWTGRFEAHLWDKSSWNSIQNKKGKQVYLGAYDSEEAAAHTYDLAALKYWGPDTILNFPAETYTKELEEMQRVTKEEYLASLRRQSSGFSRGVSKYRGVARHHHNGRWEARIGRVFGNKYLYLGTYNTQEEAAAAYDMAAIEYRGANAVTNFDISCYLDQPQLLAQLQQGPQVVPALQEELQHDVQHDLQNDNAVQELNSGEVQMPGAMDEPIALDDSTECINTPFEFDFSVEENLWSPCMDYELDAILGNNTSNSANMNEWFNDSTFESNIGCLFEGCSNIDDCSSSKHCADLAAFDFFKEGDDNDFSNMEMEITPQANDVSCPPNDVSCPPKMITVCN |
| At:As:At | MKKRLTTSTCSSSPSSSVSSSTTTSSPIQSEAPRPKRAKRAKKSSPSGDKSHNPTSPASTRRSSIYRGVTRHRWTGRFEAHLWDKNCFTSVQNKKKGRQVYLGAYDTEDAAARAYDLAALKYWGSETILNFSVEDYAKEMPEMEAVSREEYLAALRRRSSGFSRGVSKYRGVARHHHNGRWEARIGRVLGNKYLYLGTFDTQEEAAKAYDLAAIEYRGANAVTNFDISNYIDRLKKKGVFPFPVNQANHQEGILVEAKQEVETREAKEEPREEVKQQYVEEPPQEEEEKEEEKAEQQEAEIVGYSEEAAVVNCCIDSSTIMEMDRCGDNNELAWNFCMMDTGFSPFLTDQNLANENPIEYPELFNELAFEDNIDFMFDDGKHECLNLENLDCCVVGRESPPSSSSPLSCLSTDSASSTTTTTTSVSCNYLV |
| At:At:As | MKKRLTTSTCSSSPSSSVSSSTTTSSPIQSEAPRPKRAKRAKKSSPSGDKSHNPTSPASTRRSSIYRGVTRHRWTGRFEAHLWDKSSWNSIQNKKGKQVYLGAYDSEEAAAHTYDLAALKYWGPDTILNFPAETYTKELEEMQRVTKEEYLASLRRQSSGFSRGVSKYRGVARHHHNGRWEARIGRVFGNKYLYLGTYNTQEEAAAAYDMAAIEYRGANAVTNFDISCYLDQPQLLAQLQQGPQVVPALQEELQHDVQHDLQNDNAVQELNSGEVQMPGAMDEPIALDDSTECINTPFEFDFSVEENLWSPCMDYELDAILGNNTSNSANMNEWFNDSTFESNIGCLFEGCSNIDDCSSSKHCADLAAFDFFKEGDDNDFSNMEMEITPQANDVSCPPNDVSCPPKMITVCN |
